# “Thyroid nodular disease and PTEN mutation in a multicentre series of children with PTEN hamartoma tumor syndrome (PHTS)”

**DOI:** 10.1007/s12020-021-02805-y

**Published:** 2021-06-28

**Authors:** Gerdi Tuli, Jessica Munarin, Alessandro Mussa, Diana Carli, Roberto Gastaldi, Paola Borgia, Maria Cristina Vigone, Marco Abbate, Giovanni Battista Ferrero, Luisa De Sanctis

**Affiliations:** 1 Department of Pediatric Endocrinology, Regina Margherita Children Hospital, Turin, Italy; 2 Department of Public Health and Pediatrics, University of Turin, Turin, Italy; 3 Department of Paediatrics Gaslini Hospital, Genova, Italy; 4 Department of Pediatrics, IRCCS San Raffaele Scientific Institute, Milan, Italy

**Keywords:** PTEN mutation, Pediatric age, Thyroid nodule, Differentiated thyroid cancer

## Abstract

**Purpose:**

To report the incidence of 4–12% of differentiated thyroid cancer (DTC) and up to 50% of benign thyroid nodular disease and to describe nodular thyroid disease in a multicentre pediatric population with PTEN mutations. Methods: Retrospective data of pediatric patients with PTEN mutations collected from tertiary Departments of Pediatric Endocrinology of Turin, Milan and Genua, Italy, in the period 2010–2020.

**Results:**

Seventeen children with PTEN mutations were recruited in the study. Thyroid involvement was present in 12/17 (70.6%) subjects, showing a multinodular struma in 6/17 (35.3%), nodules with benign ultrasound features in 5/17 (29.4%) and a follicular adenoma in 1/17 (6%). No correlation was found between thyroid disease and gender, puberty, vascular manifestations, delayed development, or brain MRI abnormalities, while multiple lipomas were associated with thyroid disease (*p* = 0.03), as was macrocephaly. Standard Deviation (SD) score head circumference was 4.35 ± 1.35 cm in subjects with thyroid disease, 3 ± 0.43 cm (*p* = 0.02) in the group without thyroid disease. Thyroid involvement was present in all subjects with mutations in exon 6 (4/4) and exon 8 (3/3) of the PTEN gene (*p* = 0.02).

**Conclusion:**

In the presented cohort, benign thyroid disorders were prevalent, with no evidence of DTC. A correlation was found between thyroid lesions and head circumference and the occurrence of multiple lipomas. Future studies in larger cohorts should assess whether risk stratification is needed when recommending surveillance strategies in children or young adolescents with PTEN hamartoma syndrome.

## Introduction

The phosphatase and tensin homolog (PTEN) gene, located on 10q23 chromosome band, is a tumor suppressor gene with a fundamental role in the molecular pathways that mediate cell proliferation, migration, and apoptosis. Germline PTEN mutations, inherited with an autosomal dominant mechanism, have been related with the etiology of “PTEN hamartoma tumor syndromes” (PHTS), which include Cowden syndrome (CS, OMIM 158350), Bannayan-Riley-Ruvalcaba syndrome (BRRS, OMIM 153480) and *PTEN-*related proteus syndrome (PS, OMIM 176920) [1–5].

BRRS is considered the PHTS form of pediatric age and is characterized by macrocephaly, benign hamartomas, pigmented glans macules, lipomas, hemangiomas, and developmental delay or the presence of an autistic trait. Other phenotypic features of BRRS disorder include prenatal or postnatal onset of high-arched palate overgrowth, macrosomia, hypotonia, joint hyperextensibility, downward slanting palpebral fissures, frontal bossing, hypoglycemia, seizures, and café au lait spots. These phenotypic features are highly variable, although they seem to cluster within the same family [1–5].

Patients with PTEN mutations are at higher risk for malignancies of the breast, endometrium, colon, kidney and thyroid. Differentiated thyroid cancer (DTC) is one of the most common types of cancer, with a lifetime risk of 35-38% [6]; the increased risk is also present in pediatric age. Several authors report an incidence of 4–12% of DTC in the pediatric population affected by PTEN mutations, mainly follicular, and up to 50% of benign nodular thyroid disease [6]. Actually, there are no data on the behavior of such tumors but a surveillance programme is absolutely needed in these patients. Most guidelines agree on the annual surveillance program from the age of 7–10, preferring to postpone the age of initiation of screening to 10 years to avoid overdiagnosis and family anxiety [6–9].

The rationale of the surveillance program is to detect thyroid cancer at an early stage, to prevent surgical complications such as hypoparathyroidism and recurrent nerve injury, and to avoid high doses of radioiodine treatment when needed.

In this paper, we describe nodular thyroid disease in a multicentre cohort of 17 patients with PTEN mutation and discuss the DTC surveillance program in childhood.

## Materials and methods

Retrospective data were collected from all pediatric patients affected by PTEN mutation who referred to the Departments of Pediatric Endocrinology of Regina Margherita Children’s Hospital of Turin, San Raffaele Hospital of Milan and Giannina Gaslini Hospital of Genua between the years 2010 and 2020.

Patients data were extracted from the electronic medical records, such as clinical features of PHTS, anthropometric data, type of PTEN gene mutation, thyroid hormone profile, thyroid ultrasound features, and cytological [based on the Italian Consensus Statement of 2014 (10)] and histological data.

Statistical analysis and graphs were performed using Graphpad 7 software (GraphPad Software, La Jolla, CA, USA) by the chi-quadrate test to compare differences between groups. The calculations were considered statistically significant when the *P* value was less than 0.05.

## Results

Seventeen children and adolescents (9 males and 8 females, age at diagnosis 7.53 ± 3.81 years old) with PTEN mutation were recruited; their clinical data are presented in Table 1. The follow up period was 4.71 ± 0.88 years.

**Table 1:** Clinical features in the study cohort of children and adolescents with a genetically confirmed PTEN mutation

No. (Sex)	Age at diagnosis	PTEN Mutation	Familiarity	Neonatal overgrowth	Macrocephaly (HC SDS)	H SDS	BMI SDS	Skin anomalies	Vascular anomalies	Delayed development	Brain MRI	Thyroid disease
1(M)	9	c.959 dup, alias c.956_957insT exon 8	+	–	+(3.4)	1.63	0.11	Multiple lipoma	–	+	Ventricular dilatation	+
2(F)	14.5	c.959 dup, alias c.956_957insT exon 8	+	–	+(2.5)	0.23	−1.51	Multiple lipoma	–	+	-	+
3(M)	11	Exon 7 deletion	–	–	+(3.1)	0.17	−1.76	–	–	+	−	−
4(M)	5	Exon 1 deletion	+	–	+(4.3)	−0.83	-0.81	Multiple lipoma	Facial angioma	+	Periventricular leucomalacia	+
5(F)	14	Exon 1 deletion	+	–	+(5.9)	−0.44	0.98	–	Sacral angioma	–	−	+
6(F)	10	Exon 1 deletion	+	+	+(3.0)	−0.91	0.27	–	–	–	−	-
7(M)	7	c.17_18delAA (p.Lys6Argfs) Exon 1	+	+	+(3.9)	2.00	0.97	Multiple lipoma	–	+	−	−
8(M)	10	c.406 T > C p.Cys136Arg Exon 5	–	–	+(3.8)	−0.20	−0.57	Multiple lipoma	–	+	−	+
9(F)	10.3	c.635-1 G > C Intron 6	–	–	+(3.6)	0.73	−0.15	–	–	+	−	+
10(F)	4	c.723 T > G, p. Phe241Leu Exon 7	–	–	+(2.4)	−0.89	0.26	–	–	–	−	−
11(M)	7.8	c.476 G > A, p.Arg159Lys Exon 7	–	+	+(5.6)	0.21	0.09	–	–	+	Periventricular leucomalacia	−
12(M)	2.7	c.697 C > T Exon 7	–	–	+(4.1)	0.56	−0.50	Multiple lipoma	–	+	Ventricular dilatation	+
13(F)	7	c.635-1 G > C Intron 6	+	–	+(4.4)	0.36	−0.69	Multiple lipoma	Supra-orbital angioma	+	−	+
14(F)	6.5	Chromosome 10 deletion	+	–	+(2.9)	0.69	−1.15	–	Subcortical teleangectasia	+	−	+
15(F)	3.3	c.634 + 2 T > G Exon 6	–	–	+(4.0)	−0.38	–1.15	Multiple lipoma	−	+	−	+
16(M)	4.6	c.546_549delAAAG Exon 6	+	+	+(7.4)	1.99	1.59	Multiple lipoma	Right foot angioma	+	−	+
17(M)	1.4	c.1836-2 A > T Exon 8	+	–	+(4.0)	1.14	0.84	Multiple lipoma	−	+	Ventricular dilatation	+

PTEN mutation involved exon 1 in 4/17 subjects (23.5%), exon 5 in 1/17 (6%), exon 6 in 4/17 (23.5%), exon 7 in 4/17 (23.5%) and exon 8 in 3/17 (17.5%); in 1/17 (6%) a deletion of chromosome 10 was detected, which included the PTEN gene. Familiarity for PTEN mutations was present in 10/17 (58.8%), while a de novo mutation was detected in the remaining 7/10 (41.2%) cases.

Anthropometric data showed macrocephaly in all cases (head circumference SDS 3.89 ± 1.52 cm), with height and BMI adequate for age and gender.

Considering the other abnormalities associated with PTEN mutation, multiple lipomas were present in 10/17 (58.8%). Vascular manifestations were found in 5/17 (29.4%), mostly represented by angiomas. Delayed development was present in nearly all children and adolescents (14/17, 82.4%), while brain MRI showed abnormalities, i.e., ventricular dilatation or periventricular leucomalacia, in 5/17 (29.4%).

Thyroid gland involvement, as shown in Table 2, was present in 12/17 (70.6%) subjects and mostly showed multinodular struma (6/17, 35.3%) or nodules with benign ultrasound features (5/17, 29.4%); follicular adenoma was present in 1/17 (6%). The mean age at diagnosis of thyroid disease was 9.43 ± 3.2 years old, with an age ranging 2.52 ± 1.56 years after the diagnosis of PHTS. There was no correlation between thyroid disease and gender or puberty, or with vascular manifestations, delayed development and brain MRI abnormalities, while multiple lipomas were associated with thyroid disease (*p* = 0.03), as well as macrocephaly. SDS head circumference was 4.35 ± 1.35 cm in subjects with thyroid disease, 3 ± 0.43 cm (*p* = 0.02) in the group without thyroid disease. Thyroid involvement was present in all subjects with mutations in exon 6 (4/4) and exon 8 (3/3) of the PTEN gene (*p* = 0.02). Changes in exon 1 showed thyroid disease in 50% of cases (2/4), while the majority of patients with exon 7 variations showed no thyroid manifestations (1/4 displayed multinodular struma).

**Table 2:** Features of thyroid disease in the study cohort of children and adolescents with a genetically confirmed PTEN mutation

No. (Sex)	Age at diagnosis	Age at diagnosis of thyroid disease	Thyroid ultrasound features	FNAB	TSH (mcUI/ml)	FT4 (pg/ml)	FT3 (pg/ml)	AbTPO/AbHTG	Treatment	Histology	Other clinical features
1(M)	6	9	Multinodular struma	TIR3a	1.24	8.9	2.56	−	TT	Adenomatous nodules	−
2(F)	14.5	14.5	Multinodular struma	−	2.2	10.1	3.12	−	−	−	−
3(M)	11	−	Normal	−	1.5	15.1	3.42	−	−	−	Prostatic hypertrophy
4(M)	5	5	Multinodular struma	−	1.3	8.2	4.17	−	−	−	Interatrial defect, Seizures
5(F)	14	14	Multinodular struma	−	1.8	14.1	3.78	−	−	−	−
6(F)	10	−	Normal	−	1.2	12.3	2.45	−	−	−	−
7(M)	7	−	Normal	−	2.5	13.4	2.6	−	−	−	−
8(M)	10	13	Two hypoechogenic bilateral nodules (<1 cm)	−	2.12	15.6	2.98	−	−	−	−
9(F)	10.3	11	Right lobe 2.5 cm nodule	TIR5	3.15	10.1	2.6	−	TT	Follicolar adenoma	−
10(F)	4	−	Normal	−	1.18	14.2	3.7	−	−	−	−
11(M)	7.8	−	Normal	−	2.75	12.8	3.6	−	−	−	IgA deficiency
12(M)	2.7	6.8	Multinodular struma	−	6.19	11.8	4.16	−	TT	Adenomatous nodules	-
13(F)	7	7	Multinodular struma	−	0.88	13.5	3.87	−	TT	Adenomatous nodules	Transitory congenital hypothyroidism
14(F)	6.5	10	Three hypoechogenic right lobe nodules (<1 cm) and one hyperchogenic left lobe nodule (<1 cm)	−	3.68	10.4	3.67	−	−	−	
15(F)	3.3	6.4	Hypoechogenic right lobe nodule (>1 cm)	TIR2	1.67	12.7	4.53	−	−	−	Eosinophilic esophagitis
16(M)	4.6	10	Two hypoechogenic right lobe nodules (>1 cm) and one hyperchogenic left lobe nodule (>1 cm)	TIR2	0.82	10	3.4	−	−	−	
17(M)	1.4	6.5	Hypoechogenic left lobe nodule (>1 cm)	TIR1	2.83	11.3	4.1	−	−	−	G6PD deficiency, Vesicoureteral reflux

Thyroid hormone profile was normal and anti-thyroid antibodies were negative in all subjects. Total thyroidectomy was performed in 4 of 17 patients (23.5%) for a major multinodular struma with nodules >1 cm (2/17) or for cytological indications (1 with TIR3A and 1 with TIR5). Three subjects showed TIR2 cytology category after FNAB. Adenomatous nodules were present in 3 cases, while follicular adenoma was detected in 1 subject.

## Discussion

Thyroid cancer surveillance programs are recommended in patients with PTEN mutations due to the well-known increased risk of developing thyroid cancer. Recent literature data report a 4–12% prevalence of thyroid cancer in subjects with PTEN mutations, consistent with a low-cost, non-invasive screening program. To avoid delayed diagnosis and prevent surgical complications, as well as high doses of radioiodine treatment for metastatic thyroid cancer, ultrasound surveillance is indicated [6–9].

Most authors have suggested that thyroid disease surveillance should begin at a very young age (7–10 years old), with an annual or biennial ultrasound analysis. In our cohort, nearly half of the subjects with nodular thyroid disease (5/12, 41.7%) were less than 7 years old, unlike what has been reported by other authors who point to thyroid nodules as a rare finding in young children with PTEN mutations [6, 7]. This finding might be explained by the fact that in most cases of our cohort the PTEN gene analysis was performed at a young age for the presence of macrocephaly. However, the natural history of nodular disease in subjects with mutated PTEN does not appear to be very aggressive, as confirmed by the lack of evidence of DTC in the mean follow-up period of nearly 5 years in our cohort. This is encouraging about the predominance of the benign nodular disease in children with PTEN mutations and therefore, as suggested in recent recommendations, the appropriate age for initiation of DTC surveillance could be 10 years old [6].

In our cohort, 70.6% of subjects had nodular thyroid disease with a 47% rate of nodules >1 cm, in agreement with literature data [6–9]. In our cohort, no age or gender differences were observed between subjects without thyroid disease and those with thyroid nodules, as well as between subjects with nodules >1 cm and <1 cm. Smith et al. in a larger cohort of 64 pediatric patients showed a significant difference in timing of diagnosis between males (10–15 years) and females (8–13 years), which corresponds with the pubertal period for both genders.

Among the various clinical features of PHTS, a possible correlation between nodular thyroid disease and head circumference and the presence of multiple lipoma emerged in the analyzed cohort. Thus, a head circumference >4 SDS might be associated with a higher risk of thyroid nodules, such as the presence of multiple lipomas with a greater likehood of nodular thyroid disease. Undoubtedly, these two possible associations, not previously reported by other authors, need to be confirmed in larger cohorts.

A possible correlation between genotype and thyroid disease was also observed in the cohort presented here, as all the subjects harboring mutations in exons 6 and 8 of the gene showed thyroid nodules. However, this finding also needs to be confirmed in larger cohorts, as no genotype-phenotype correlation between PTEN mutations and thyroid disease has been reported so far [6–9].

Among nodular thyroid disease, the presence of multinodular struma was the most common finding (35.3%), followed by benign nodular disease (29.4%). No malignant nodules were detected in our cohort and fine needle biopsy (FNAB) was performed only in case of nodules >1 cm or when ultrasound features were highly suspicious of malignancy. Total thyroidectomy was chosen in all cases of surgery; cytological analysis showed adenomatous nodules in 3 cases and follicular adenoma in 1 female subject. We agree with the recommendation of other groups that in these patients, when surgery is performed, the total thyroidectomy approach is the most advisable option in pediatric age [6–9].

All the subjects we collected had a normal thyroid hormone profile and none showed anti-peroxidase or anti-thyroglobulin antibodies, as reported by many authors [6–9].

We are aware that the limitations of this study are the retrospective nature of the data collection and the small size of the cohort, which limit the strength of the statistical analysis.

Therefore, the association of the severity of macrocephaly and multiple lipomas with nodular thyroid disease needs to be confirmed in larger cohorts, as well as the involvement of exon 6 and 8, which appears to increase the risk of nodular disease.

In conclusion, nodular thyroid disease is often the first serious complication in children with PTEN mutations, who have a higher risk of thyroid cancer. Therefore, an ultrasound surveillance program is recommended in all patients from infancy to avoid delayed diagnosis. The frequency of ultrasound screening should be decided on the basis of the first evaluation after confirmation of the PTEN mutation, but annual monitoring may be proposed, especially if severe macrocephaly is present. The proposed interval has to be modified and adapted when suspicious features of malignancy are present. In these cases, a fine needle biopsy is recommended and, if surgery is required, total thyroidectomy should be preferred.
